# Bis[bis­(diphenyl­phosphino­yl)acetonitrile-κ^2^ *O*,*O*′]copper(II)

**DOI:** 10.1107/S1600536811031564

**Published:** 2011-08-11

**Authors:** Sicelo V. Sithole, Richard J. Staples, Werner E. Van Zyl

**Affiliations:** a School of Chemistry, University of KwaZulu-Natal, Westville Campus, Private Bag X54001, Durban 4000, South Africa; b Department of Chemistry, Michigan State University, East Lansing, MI 48824-1322, USA

## Abstract

The title complex, [Cu(C_26_H_20_NO_2_P_2_)_2_], contains a central Cu^II^ atom surrounded by two homoleptic bidentate ligands, which form two five-membered chelate rings. The Cu atom binds to four O atoms, resulting in a four-coordinate square-planar complex. The asymmetric unit contains half of the complex, the other half being completed by inversion symmetry. The Cu—O bond lengths have similar distances, *viz.* 1.9153 (10) Å for the pair opposite (*trans*) each other and 1.9373 (10) Å for the other (*trans*) pair. The P—O bond lengths are 1.5250 (11) Å, indicating significant electron delocalization across the O—P—C—P—O atoms in the chelate ring, resulting in a longer P—O bond length when compared to a formal double-bond P=O character (much shorter at approximately 1.47 Å). The two inter­secting O—Cu—O angles are both linear at 180°, whilst the remaining L-shaped O—Cu—O bond angles are 88.26 (5) and 91.74 (5)°. The C—C N fragment is slightly distorted from linearity at 177.44 (19)° and the C N bond length of 1.151 (2) Å indicates predominantly triple-bond character.

## Related literature

For recent work on bis­(diphenyl­phosphane)acetonitrile, see: Braun *et al.* (2007 ▶); Spannhoff *et al.* (2009 ▶). For a bis­(di­phen­yl­phosphane)acetonitrile complex of gold(I), see: Sithole *et al.* (2011 ▶) and for bis­(diphenyl­phosphane)acetonitrile oxides and sulfides (and their lithia­ted compounds), see: Braun *et al.* (2008 ▶). For background to our inter­est in dinuclear gold(I) complexes, see: Van Zyl (2010 ▶)
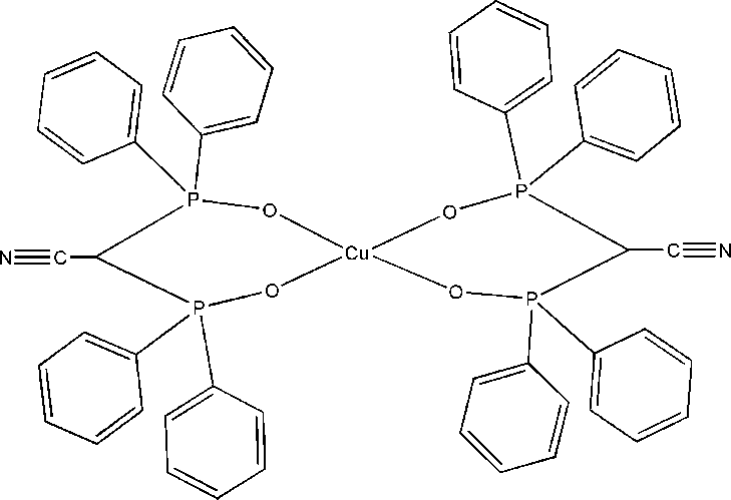


## Experimental

### 

#### Crystal data

[Cu(C_26_H_20_NO_2_P_2_)_2_]
*M* *_r_* = 944.28
Monoclinic, 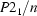

*a* = 9.5917 (7) Å
*b* = 25.8793 (19) Å
*c* = 9.6648 (7) Å
β = 111.526 (1)°
*V* = 2231.7 (3) Å^3^
*Z* = 2
Mo *K*α radiation
μ = 0.68 mm^−1^
*T* = 173 K
0.33 × 0.23 × 0.12 mm

#### Data collection

Bruker APEXII CCD diffractometer
Absorption correction: multi-scan (*SADABS*; Bruker, 2009 ▶) *T*_min_ = 0.807, *T*_max_ = 0.920
43689 measured reflections
5640 independent reflections
4832 reflections with *I* > 2σ(*I*)
*R*_int_ = 0.036

#### Refinement

*R*[*F* ^2^ > 2σ(*F* ^2^)] = 0.033
*wR*(*F* ^2^) = 0.086
*S* = 1.03
5640 reflections
286 parameters
H-atom parameters constrained
Δρ_max_ = 0.49 e Å^−3^
Δρ_min_ = −0.32 e Å^−3^

### 

Data collection: *COSMO* (Bruker, 2009 ▶); cell refinement: *APEX2* (Bruker, 2009 ▶); data reduction: *SAINT* (Bruker, 2009 ▶); program(s) used to solve structure: *SHELXS97* (Sheldrick, 2008 ▶); program(s) used to refine structure: *SHELXL97* (Sheldrick, 2008 ▶); molecular graphics: *SHELXTL* (Sheldrick, 2008 ▶); software used to prepare material for publication: *SHELXTL*.

## Supplementary Material

Crystal structure: contains datablock(s) I, global. DOI: 10.1107/S1600536811031564/ff2023sup1.cif

Structure factors: contains datablock(s) I. DOI: 10.1107/S1600536811031564/ff2023Isup2.hkl

Additional supplementary materials:  crystallographic information; 3D view; checkCIF report

## Figures and Tables

**Fig. 1.**
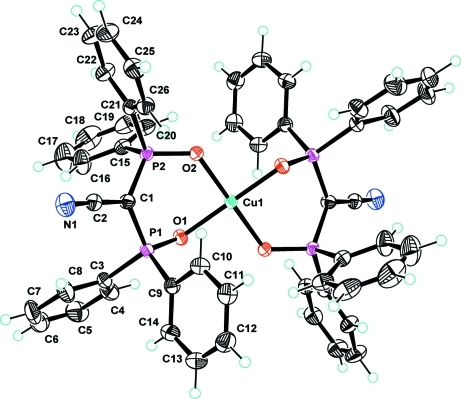
Molecular structure of the title complex. Ellipsoids are drawn at the 50% probability level. Unlabelled atoms are related to the reference ones by the (-x -y, 1-z) symmetry transformation.

## supplementary crystallographic information

### Comment

The chemistry of the ligand bis(diphenylphosphane)acetonitrile, (dppm-CN), was recently revived when a facile preparation thereof was reported, starting with readily available acetonitrile (Braun *et al.*, 2007). Development of its chemistry followed thereafter, including the observed sharp increase in acidity by the replacement of a proton with a cyano group on the bridging carbon atom (Spannhoff *et al.*, 2009, and references therein). In continuing our interest in dinuclear gold(I) complexes (Van Zyl, 2010), we recently reported the first structural investigation of a chlorogold(I) complex with the ligand dppm-CN, (Sithole *et al.*, 2011).

A relatively straightforward method for the preparation of complex (I) would entail the reaction between Li[N≡C—C(Ph_2_P=O)_2_], prepared as described (Braun *et al.*, 2008), and CuCl_2_ or Cu(NO_3_)_2_ (molar ratio 2:1), leading to formation of complex (I) after removal of LiCl or LiNO_3_. However, in the present study, we report complex (I) was obtained in a surprisingly different manner. Formation of (I) occurred post-synthesis during the crystallization process. An initial reaction between the well characterized complexes [Au_2_{dppm-CN}_2_], and [Cu(CH_3_CN)_4_][PF_6_] (1:1 molar ratio) was performed in an attempt to coordinate the copper(I) center to the CN group. The Experimental section describes a complex formed and isolated from this reaction as a colorless, free-flowing powder and suggests no sign of metal oxidation (both Au(I) and Cu(I) are closed-shell *d^10^* systems and are usually colorless or pale yellow compounds, hence any sign of oxidation leading to deep color changes for either metal is readily observed). However, during subsequent crystallization procedures obvious oxidation occurred, firstly at the metal center where the precursor colorless Cu(I) species oxidized to a green-colored Cu(II) complex. Secondly, the P atoms in the anionic [dppm-CN]¯ ligand did oxidize to the corresponding oxide analogue (not uncommon in the presence of air or moisture), and the ligand transferred also from the gold(I) center to the much more oxophillic Cu(II) center whilst retaining the negative anionic charge, *i.e.* it did not become protonated and thus neutral in the process. No attempt was made in this study to determine the concurrent reduced species, but presumably the Au(I) species reduced to Au(0) metal. Single crystal X-ray analysis subsequently revealed the green colored material to be identified as complex (I).

### Experimental

Preparation and characterization of complex obtained pre-crystallization: A Schlenk flask equipped with a magnetic stirrer bar was charged with previously prepared [Au_2_{dppm-CN}_2_] complex (400 mg, 0.33 mmol) dissolved in dichloromethane (5 ml). A dichloromethane (8 ml) solution of [Cu(CH_3_CN)_4_][PF_6_] (123 mg, 0.33 mmol) was then added dropwise to the gold-containing solution at room temperature. The solution turned cloudy and was stirred for an hour, after which all dichloromethane solvent was removed under reduced pressure. Dry Et_2_O (2 *x* 2 ml) was added to wash the product which was further dried in *vacuo* for 2 hrs. The product was obtained as a free flowing off-white powder. Yield: (387 mg, 0.21 mmol) 65%; Mp: 155–160 *^o^*C (decompose); ^1^H NMR (400 MHz, CDCl_3_, 298 K) *δ*_H_= 7.68–7.53 (m, 40H, Ph); ^31^P NMR (101 MHz, CDCl_3_, 298 K) *δ*_P_ = 22.8 (s, 2P). Green single crystals were obtained by slow evaporation of a dichloromethane solution of the complex.

### Refinement

All non-hydrogen atoms are refined anisotropically. H atoms were calculated by geometrical methods and refined as a riding model, with C—H distace 0.95 Å and *U*_iso_(H) = 1.2*U*_eq_(C). The crystal used for the diffraction study showed no decomposition during data collection.

### Crystal data

**Table:**

[Cu(C_26_H_20_NO_2_P_2_)_2_]	*F*(000) = 974
*M**_r_* = 944.28	*D*_x_ = 1.405 Mg m^−^^3^
Monoclinic, *P*2_1_/*n*	Mo *K*α radiation, λ = 0.71073 Å
Hall symbol: -P 2yn	Cell parameters from 9929 reflections
*a* = 9.5917 (7) Å	θ = 2.4–28.7°
*b* = 25.8793 (19) Å	µ = 0.68 mm^−^^1^
*c* = 9.6648 (7) Å	*T* = 173 K
β = 111.526 (1)°	Block, green
*V* = 2231.7 (3) Å^3^	0.33 × 0.23 × 0.12 mm
*Z* = 2	

### Data collection

**Table:**

Bruker APEXII CCD diffractometer	5640 independent reflections
Radiation source: fine-focus sealed tube	4832 reflections with *I* > 2σ(*I*)
graphite	*R*_int_ = 0.036
Detector resolution: 836.6 pixels mm^-1^	θ_max_ = 29.1°, θ_min_ = 2.4°
ω,and/f 0.5° scans	*h* = −12→12
Absorption correction: multi-scan (*SADABS*; Bruker, 2009)	*k* = −34→34
*T*_min_ = 0.807, *T*_max_ = 0.920	*l* = −13→13
43689 measured reflections	

### Refinement

**Table:**

Refinement on *F*^2^	Primary atom site location: structure-invariant direct methods
Least-squares matrix: full	Secondary atom site location: difference Fourier map
*R*[*F*^2^ > 2σ(*F*^2^)] = 0.033	Hydrogen site location: inferred from neighbouring sites
*wR*(*F*^2^) = 0.086	H-atom parameters constrained
*S* = 1.03	*w* = 1/[σ^2^(*F*_o_^2^) + (0.0428*P*)^2^ + 1.1569*P*] where *P* = (*F*_o_^2^ + 2*F*_c_^2^)/3
5640 reflections	(Δ/σ)_max_ = 0.001
286 parameters	Δρ_max_ = 0.49 e Å^−^^3^
0 restraints	Δρ_min_ = −0.32 e Å^−^^3^

### Special details

**Table:**

Experimental. Data was collected using a BRUKER CCD (charge coupled device) based diffractometer equipped with an Oxford low-temperature apparatus operating at 173 K. A suitable crystal was chosen and mounted on a glass fiber or nylon loop using Paratone oil for Mo radiation and Mineral oil for Copper radiation. Data were measured using omega and phi scans of 0.5° per frame for 20 s. The total number of images were based on results from the program COSMO where redundancy was expected to be 4 and completeness to 0.83Å to 100%. Cell parameters were retrieved using *APEX* II software and refined using *SAINT* on all observed reflections.Data reduction was performed using the *SAINT* software which corrects for Lp. Scaling and absorption corrections were applied using *SADABS6* multi-scan technique, supplied by George Sheldrick. The structures are solved by the direct method using the *SHELXS97* program and refined by least squares method on F2, *SHELXL97*, incorporated in *SHELXTL*-PC V 6.14.All H atoms were placed in calculated positions and refined using a riding model. C—H(aromatic) = 0.94 Å and *U*_iso_(H) = 1.2Ueq(C) C—H (alaphatic) = 0.99 Å and *U*_iso_(H) = 1.2Ueq(C) CH2 = 0.98 Å and *U*_iso_(H) = 1.2Ueq(C) CH3 = 0.97Å and *U*_iso_(H) = 1.5Ueq(C) N—H = 0.86 (0.92)Å and *U*_iso_(H) = 1.2 *U*_eq_(N) O—H(alcohol) = 0.85Åand *U*_iso_(H) = 1.2Ueq(O) O—H(acid) = 0.82 Å and *U*_iso_(H) = 1.5Ueq(O)
Geometry. All e.s.d.'s (except the e.s.d. in the dihedral angle between two l.s. planes) are estimated using the full covariance matrix. The cell e.s.d.'s are taken into account individually in the estimation of e.s.d.'s in distances, angles and torsion angles; correlations between e.s.d.'s in cell parameters are only used when they are defined by crystal symmetry. An approximate (isotropic) treatment of cell e.s.d.'s is used for estimating e.s.d.'s involving l.s. planes.
Refinement. Refinement of *F*^2^ against ALL reflections. The weighted *R*-factor *wR* and goodness of fit *S* are based on *F*^2^, conventional *R*-factors *R* are based on *F*, with *F* set to zero for negative *F*^2^. The threshold expression of *F*^2^ > σ(*F*^2^) is used only for calculating *R*-factors(gt) *etc*. and is not relevant to the choice of reflections for refinement. *R*-factors based on *F*^2^ are statistically about twice as large as those based on *F*, and *R*- factors based on ALL data will be even larger.

### Fractional atomic coordinates and isotropic or equivalent isotropic displacement parameters (Å^2^)

**Table:**

	*x*	*y*	*z*	*U*_iso_*/*U*_eq_	
Cu1	0.0000	0.0000	0.5000	0.01782 (8)	
P1	0.11652 (4)	0.085542 (15)	0.73438 (4)	0.02022 (9)	
P2	0.19258 (4)	0.090614 (15)	0.46718 (4)	0.01886 (9)	
O1	−0.01591 (12)	0.05965 (4)	0.61444 (12)	0.0222 (2)	
O2	0.12911 (12)	0.03636 (4)	0.42218 (12)	0.0230 (2)	
N1	0.49225 (17)	0.14605 (6)	0.81575 (19)	0.0374 (4)	
C1	0.24792 (17)	0.10182 (6)	0.65683 (17)	0.0217 (3)	
C2	0.38298 (18)	0.12620 (6)	0.74163 (18)	0.0243 (3)	
C3	0.04936 (19)	0.14124 (6)	0.80190 (19)	0.0259 (3)	
C4	−0.1033 (2)	0.15090 (8)	0.7557 (2)	0.0349 (4)	
H4	−0.1730	0.1273	0.6911	0.042*	
C5	−0.1545 (3)	0.19521 (8)	0.8038 (3)	0.0474 (5)	
H5	−0.2590	0.2017	0.7729	0.057*	
C6	−0.0534 (3)	0.22945 (8)	0.8963 (3)	0.0525 (6)	
H6	−0.0884	0.2598	0.9284	0.063*	
C7	0.0989 (3)	0.22001 (9)	0.9432 (3)	0.0571 (7)	
H7	0.1680	0.2438	1.0074	0.069*	
C8	0.1504 (2)	0.17605 (8)	0.8966 (3)	0.0442 (5)	
H8	0.2550	0.1695	0.9291	0.053*	
C9	0.19949 (18)	0.04236 (6)	0.88816 (18)	0.0241 (3)	
C10	0.2915 (2)	0.00309 (7)	0.87194 (19)	0.0289 (4)	
H10	0.3206	0.0028	0.7880	0.035*	
C11	0.3408 (2)	−0.03571 (7)	0.9783 (2)	0.0336 (4)	
H11	0.4036	−0.0625	0.9670	0.040*	
C12	0.2983 (2)	−0.03529 (8)	1.1009 (2)	0.0356 (4)	
H12	0.3309	−0.0621	1.1729	0.043*	
C13	0.2088 (3)	0.00393 (8)	1.1186 (2)	0.0389 (4)	
H13	0.1812	0.0044	1.2035	0.047*	
C14	0.1591 (2)	0.04268 (7)	1.0126 (2)	0.0335 (4)	
H14	0.0972	0.0696	1.0249	0.040*	
C15	0.05405 (18)	0.13679 (6)	0.36258 (18)	0.0248 (3)	
C16	0.0541 (2)	0.18605 (7)	0.4181 (3)	0.0440 (5)	
H16	0.1279	0.1955	0.5112	0.053*	
C17	−0.0536 (3)	0.22178 (9)	0.3379 (3)	0.0625 (7)	
H17	−0.0526	0.2558	0.3750	0.075*	
C18	−0.1616 (3)	0.20742 (10)	0.2045 (3)	0.0596 (7)	
H18	−0.2361	0.2316	0.1505	0.072*	
C19	−0.1631 (2)	0.15861 (10)	0.1484 (2)	0.0463 (5)	
H19	−0.2383	0.1493	0.0561	0.056*	
C20	−0.05537 (19)	0.12307 (8)	0.22608 (19)	0.0316 (4)	
H20	−0.0557	0.0894	0.1868	0.038*	
C21	0.35557 (17)	0.09861 (6)	0.42016 (17)	0.0226 (3)	
C22	0.3618 (2)	0.13313 (7)	0.3125 (2)	0.0322 (4)	
H22	0.2780	0.1543	0.2610	0.039*	
C23	0.4920 (2)	0.13646 (9)	0.2805 (2)	0.0445 (5)	
H23	0.4968	0.1599	0.2069	0.053*	
C24	0.6140 (2)	0.10571 (8)	0.3557 (2)	0.0406 (5)	
H24	0.7030	0.1085	0.3347	0.049*	
C25	0.6072 (2)	0.07084 (8)	0.4614 (2)	0.0344 (4)	
H25	0.6909	0.0494	0.5118	0.041*	
C26	0.47879 (18)	0.06714 (7)	0.49360 (19)	0.0272 (3)	
H26	0.4743	0.0431	0.5661	0.033*	

### Atomic displacement parameters (Å^2^)

**Table:**

	*U*^11^	*U*^22^	*U*^33^	*U*^12^	*U*^13^	*U*^23^
Cu1	0.01623 (13)	0.01786 (13)	0.01979 (13)	−0.00193 (9)	0.00712 (10)	−0.00220 (9)
P1	0.02000 (19)	0.02058 (19)	0.02087 (19)	−0.00208 (14)	0.00841 (15)	−0.00352 (14)
P2	0.01714 (18)	0.01878 (18)	0.02072 (19)	−0.00176 (14)	0.00704 (15)	−0.00045 (14)
O1	0.0191 (5)	0.0225 (5)	0.0259 (6)	−0.0019 (4)	0.0093 (4)	−0.0054 (4)
O2	0.0263 (6)	0.0213 (5)	0.0259 (6)	−0.0055 (4)	0.0148 (5)	−0.0042 (4)
N1	0.0250 (8)	0.0389 (9)	0.0417 (9)	−0.0058 (6)	0.0046 (7)	−0.0069 (7)
C1	0.0187 (7)	0.0261 (7)	0.0206 (7)	−0.0035 (6)	0.0075 (6)	−0.0018 (6)
C2	0.0233 (8)	0.0237 (7)	0.0255 (8)	0.0009 (6)	0.0086 (6)	0.0004 (6)
C3	0.0324 (9)	0.0210 (7)	0.0281 (8)	0.0001 (6)	0.0157 (7)	−0.0031 (6)
C4	0.0348 (10)	0.0355 (10)	0.0347 (10)	0.0061 (8)	0.0130 (8)	−0.0032 (8)
C5	0.0535 (13)	0.0428 (11)	0.0518 (13)	0.0210 (10)	0.0262 (11)	0.0052 (10)
C6	0.0843 (18)	0.0255 (9)	0.0676 (15)	0.0096 (10)	0.0512 (14)	−0.0018 (10)
C7	0.0759 (17)	0.0362 (11)	0.0741 (17)	−0.0183 (11)	0.0450 (14)	−0.0285 (11)
C8	0.0405 (11)	0.0404 (11)	0.0563 (13)	−0.0104 (9)	0.0231 (10)	−0.0226 (10)
C9	0.0243 (8)	0.0250 (8)	0.0229 (7)	−0.0035 (6)	0.0084 (6)	−0.0024 (6)
C10	0.0286 (9)	0.0334 (9)	0.0257 (8)	0.0013 (7)	0.0113 (7)	0.0007 (7)
C11	0.0298 (9)	0.0327 (9)	0.0353 (9)	0.0039 (7)	0.0085 (7)	0.0027 (7)
C12	0.0378 (10)	0.0360 (9)	0.0263 (9)	−0.0029 (8)	0.0039 (7)	0.0057 (7)
C13	0.0523 (12)	0.0420 (11)	0.0253 (9)	−0.0019 (9)	0.0177 (8)	0.0005 (8)
C14	0.0424 (10)	0.0330 (9)	0.0298 (9)	0.0018 (8)	0.0187 (8)	−0.0023 (7)
C15	0.0210 (7)	0.0271 (8)	0.0262 (8)	0.0006 (6)	0.0085 (6)	0.0041 (6)
C16	0.0452 (11)	0.0274 (9)	0.0499 (12)	0.0070 (8)	0.0062 (10)	0.0019 (8)
C17	0.0715 (17)	0.0327 (11)	0.0784 (18)	0.0211 (11)	0.0216 (14)	0.0119 (11)
C18	0.0472 (13)	0.0583 (15)	0.0683 (16)	0.0239 (11)	0.0151 (12)	0.0355 (13)
C19	0.0283 (10)	0.0674 (15)	0.0378 (11)	0.0030 (9)	0.0055 (8)	0.0239 (10)
C20	0.0244 (8)	0.0437 (10)	0.0256 (8)	−0.0016 (7)	0.0077 (7)	0.0058 (7)
C21	0.0211 (7)	0.0237 (7)	0.0236 (7)	−0.0037 (6)	0.0090 (6)	−0.0034 (6)
C22	0.0300 (9)	0.0331 (9)	0.0369 (10)	−0.0013 (7)	0.0162 (8)	0.0069 (7)
C23	0.0454 (12)	0.0495 (12)	0.0491 (12)	−0.0050 (9)	0.0299 (10)	0.0114 (10)
C24	0.0314 (10)	0.0491 (11)	0.0514 (12)	−0.0077 (8)	0.0269 (9)	−0.0045 (9)
C25	0.0231 (8)	0.0397 (10)	0.0422 (10)	0.0016 (7)	0.0141 (8)	−0.0061 (8)
C26	0.0239 (8)	0.0287 (8)	0.0293 (8)	−0.0006 (6)	0.0102 (7)	−0.0001 (7)

### Geometric parameters (Å, °)

**Table:**

Cu1—O2	1.9153 (10)	C11—C12	1.388 (3)
Cu1—O2^i^	1.9153 (10)	C11—H11	0.9500
Cu1—O1^i^	1.9373 (10)	C12—C13	1.380 (3)
Cu1—O1	1.9373 (10)	C12—H12	0.9500
P1—O1	1.5250 (11)	C13—C14	1.387 (3)
P1—C1	1.7381 (16)	C13—H13	0.9500
P1—C9	1.7953 (17)	C14—H14	0.9500
P1—C3	1.7963 (16)	C15—C16	1.383 (3)
P2—O2	1.5287 (11)	C15—C20	1.397 (2)
P2—C1	1.7353 (16)	C16—C17	1.391 (3)
P2—C21	1.7929 (16)	C16—H16	0.9500
P2—C15	1.7972 (16)	C17—C18	1.376 (4)
N1—C2	1.151 (2)	C17—H17	0.9500
C1—C2	1.403 (2)	C18—C19	1.373 (4)
C3—C4	1.388 (2)	C18—H18	0.9500
C3—C8	1.392 (3)	C19—C20	1.381 (3)
C4—C5	1.393 (3)	C19—H19	0.9500
C4—H4	0.9500	C20—H20	0.9500
C5—C6	1.374 (3)	C21—C22	1.389 (2)
C5—H5	0.9500	C21—C26	1.396 (2)
C6—C7	1.383 (4)	C22—C23	1.395 (3)
C6—H6	0.9500	C22—H22	0.9500
C7—C8	1.380 (3)	C23—C24	1.381 (3)
C7—H7	0.9500	C23—H23	0.9500
C8—H8	0.9500	C24—C25	1.382 (3)
C9—C14	1.392 (2)	C24—H24	0.9500
C9—C10	1.392 (2)	C25—C26	1.380 (2)
C10—C11	1.390 (2)	C25—H25	0.9500
C10—H10	0.9500	C26—H26	0.9500
			
O2—Cu1—O2^i^	180.0	C12—C11—C10	119.92 (17)
O2—Cu1—O1^i^	88.26 (5)	C12—C11—H11	120.0
O2^i^—Cu1—O1^i^	91.74 (5)	C10—C11—H11	120.0
O2—Cu1—O1	91.74 (5)	C13—C12—C11	120.17 (17)
O2^i^—Cu1—O1	88.26 (5)	C13—C12—H12	119.9
O1^i^—Cu1—O1	180.0	C11—C12—H12	119.9
O1—P1—C1	108.19 (7)	C12—C13—C14	120.08 (18)
O1—P1—C9	110.39 (7)	C12—C13—H13	120.0
C1—P1—C9	109.66 (8)	C14—C13—H13	120.0
O1—P1—C3	108.61 (7)	C13—C14—C9	120.30 (18)
C1—P1—C3	112.06 (8)	C13—C14—H14	119.9
C9—P1—C3	107.93 (8)	C9—C14—H14	119.9
O2—P2—C1	112.92 (7)	C16—C15—C20	119.59 (17)
O2—P2—C21	109.10 (7)	C16—C15—P2	119.98 (14)
C1—P2—C21	106.93 (7)	C20—C15—P2	120.42 (13)
O2—P2—C15	108.40 (7)	C15—C16—C17	120.1 (2)
C1—P2—C15	111.07 (8)	C15—C16—H16	119.9
C21—P2—C15	108.31 (8)	C17—C16—H16	119.9
P1—O1—Cu1	124.47 (6)	C18—C17—C16	119.5 (2)
P2—O2—Cu1	125.94 (6)	C18—C17—H17	120.3
C2—C1—P2	123.44 (12)	C16—C17—H17	120.3
C2—C1—P1	121.15 (12)	C19—C18—C17	120.9 (2)
P2—C1—P1	115.20 (9)	C19—C18—H18	119.5
N1—C2—C1	177.44 (19)	C17—C18—H18	119.5
C4—C3—C8	119.55 (16)	C18—C19—C20	120.0 (2)
C4—C3—P1	120.28 (13)	C18—C19—H19	120.0
C8—C3—P1	120.13 (14)	C20—C19—H19	120.0
C3—C4—C5	120.01 (19)	C19—C20—C15	119.80 (19)
C3—C4—H4	120.0	C19—C20—H20	120.1
C5—C4—H4	120.0	C15—C20—H20	120.1
C6—C5—C4	119.8 (2)	C22—C21—C26	119.65 (15)
C6—C5—H5	120.1	C22—C21—P2	123.05 (13)
C4—C5—H5	120.1	C26—C21—P2	117.27 (12)
C5—C6—C7	120.49 (19)	C21—C22—C23	119.59 (17)
C5—C6—H6	119.8	C21—C22—H22	120.2
C7—C6—H6	119.8	C23—C22—H22	120.2
C8—C7—C6	120.0 (2)	C24—C23—C22	120.19 (18)
C8—C7—H7	120.0	C24—C23—H23	119.9
C6—C7—H7	120.0	C22—C23—H23	119.9
C7—C8—C3	120.1 (2)	C23—C24—C25	120.25 (17)
C7—C8—H8	120.0	C23—C24—H24	119.9
C3—C8—H8	120.0	C25—C24—H24	119.9
C14—C9—C10	119.37 (16)	C26—C25—C24	120.03 (18)
C14—C9—P1	122.14 (13)	C26—C25—H25	120.0
C10—C9—P1	117.91 (13)	C24—C25—H25	120.0
C11—C10—C9	120.14 (17)	C25—C26—C21	120.27 (17)
C11—C10—H10	119.9	C25—C26—H26	119.9
C9—C10—H10	119.9	C21—C26—H26	119.9
			
C1—P1—O1—Cu1	54.82 (10)	C1—P1—C9—C14	146.86 (15)
C9—P1—O1—Cu1	−65.18 (10)	C3—P1—C9—C14	24.52 (17)
C3—P1—O1—Cu1	176.68 (8)	O1—P1—C9—C10	77.18 (14)
O2—Cu1—O1—P1	−59.93 (8)	C1—P1—C9—C10	−41.93 (15)
O2^i^—Cu1—O1—P1	120.07 (8)	C3—P1—C9—C10	−164.26 (13)
O1^i^—Cu1—O1—P1	−147 (6)	C14—C9—C10—C11	0.8 (3)
C1—P2—O2—Cu1	40.14 (11)	P1—C9—C10—C11	−170.67 (14)
C21—P2—O2—Cu1	158.90 (8)	C9—C10—C11—C12	0.0 (3)
C15—P2—O2—Cu1	−83.36 (10)	C10—C11—C12—C13	−0.9 (3)
O2^i^—Cu1—O2—P2	−122 (9)	C11—C12—C13—C14	1.0 (3)
O1^i^—Cu1—O2—P2	−174.51 (9)	C12—C13—C14—C9	−0.2 (3)
O1—Cu1—O2—P2	5.49 (9)	C10—C9—C14—C13	−0.7 (3)
O2—P2—C1—C2	135.75 (13)	P1—C9—C14—C13	170.41 (15)
C21—P2—C1—C2	15.74 (16)	O2—P2—C15—C16	154.64 (15)
C15—P2—C1—C2	−102.24 (15)	C1—P2—C15—C16	30.03 (18)
O2—P2—C1—P1	−49.44 (11)	C21—P2—C15—C16	−87.11 (17)
C21—P2—C1—P1	−169.45 (9)	O2—P2—C15—C20	−24.48 (16)
C15—P2—C1—P1	72.57 (11)	C1—P2—C15—C20	−149.10 (14)
O1—P1—C1—C2	−179.75 (13)	C21—P2—C15—C20	93.76 (15)
C9—P1—C1—C2	−59.30 (15)	C20—C15—C16—C17	−0.4 (3)
C3—P1—C1—C2	60.54 (16)	P2—C15—C16—C17	−179.55 (19)
O1—P1—C1—P2	5.31 (11)	C15—C16—C17—C18	1.2 (4)
C9—P1—C1—P2	125.76 (9)	C16—C17—C18—C19	−1.0 (4)
C3—P1—C1—P2	−114.40 (10)	C17—C18—C19—C20	0.1 (4)
P2—C1—C2—N1	178 (100)	C18—C19—C20—C15	0.7 (3)
P1—C1—C2—N1	4(4)	C16—C15—C20—C19	−0.5 (3)
O1—P1—C3—C4	6.89 (17)	P2—C15—C20—C19	178.61 (14)
C1—P1—C3—C4	126.36 (15)	O2—P2—C21—C22	113.92 (15)
C9—P1—C3—C4	−112.80 (15)	C1—P2—C21—C22	−123.65 (15)
O1—P1—C3—C8	−170.65 (15)	C15—P2—C21—C22	−3.87 (17)
C1—P1—C3—C8	−51.19 (18)	O2—P2—C21—C26	−64.13 (14)
C9—P1—C3—C8	69.66 (17)	C1—P2—C21—C26	58.30 (14)
C8—C3—C4—C5	0.1 (3)	C15—P2—C21—C26	178.08 (13)
P1—C3—C4—C5	−177.50 (15)	C26—C21—C22—C23	−0.9 (3)
C3—C4—C5—C6	0.5 (3)	P2—C21—C22—C23	−178.91 (15)
C4—C5—C6—C7	−0.6 (3)	C21—C22—C23—C24	−0.1 (3)
C5—C6—C7—C8	0.3 (4)	C22—C23—C24—C25	1.0 (3)
C6—C7—C8—C3	0.3 (4)	C23—C24—C25—C26	−0.9 (3)
C4—C3—C8—C7	−0.4 (3)	C24—C25—C26—C21	−0.2 (3)
P1—C3—C8—C7	177.14 (18)	C22—C21—C26—C25	1.1 (3)
O1—P1—C9—C14	−94.03 (15)	P2—C21—C26—C25	179.18 (14)

Symmetry codes: (i) −*x*, −*y*, −*z*+1.
